# Organotypic Models for Functional Drug Testing of Human Cancers

**DOI:** 10.34133/bmef.0022

**Published:** 2023-06-16

**Authors:** Yu Ling Huang, Lindsay K. Dickerson, Heidi Kenerson, Xiuyun Jiang, Venu Pillarisetty, Qiang Tian, Leroy Hood, Taranjit S. Gujral, Raymond S. Yeung

**Affiliations:** ^1^ Division of Human Biology, Fred Hutchinson Cancer Center, Seattle, WA, USA.; ^2^ Department of Surgery, University of Washington, Seattle, WA, USA.; ^3^ National Research Center for Translational Medicine, Ruijin Hospital Affiliated to Shanghai Jiao Tong University School of Medicine, Shanghai, China.; ^4^ Institute for Systems Biology, Phenome Health Institute, Seattle, WA, USA.

## Abstract

In the era of personalized oncology, there have been accelerated efforts to develop clinically relevant platforms to test drug sensitivities of individual cancers. An ideal assay will serve as a diagnostic companion to inform the oncologist of the various treatments that are sensitive and insensitive, thus improving outcome while minimizing unnecessary toxicities and costs. To date, no such platform exists for clinical use, but promising approaches are on the horizon that take advantage of improved techniques in creating human cancer models that encompass the entire tumor microenvironment, alongside technologies for assessing and analyzing tumor response. This review summarizes a number of current strategies that make use of intact human cancer tissues as organotypic cultures in drug sensitivity testing.

## Introduction

There is a growing demand for the use of human-derived tumor models in the era of precision oncology. The phenomenon of inter- and intratumor heterogeneity underlies the need for “personalized” models that reflect the cellular and molecular complexities of individual cancers, which impact tumor biology and response to treatment. Traditional preclinical cancer models such as cell lines and genetically engineered mouse models are inadequate to represent the diversity of human cancers, and thus, drug development using these tools has limited clinical application. Similarly, in vitro testing of patient-derived tumor cultures has been met with limited success, perhaps because initial models were not representative of the multitude of elements and intricate networks that comprise the tumor microenvironment (TME). With advancing technology, there has been a concerted effort to revitalize the use of fresh human cancer tissues as avatars of the disease. In this review, we will examine the role of organotypic tumor models as a tool for drug discovery and testing and contrast it with other human cancer models. We will highlight their uses in cancer research and potential utility in clinical care.

**Fig. 1.**
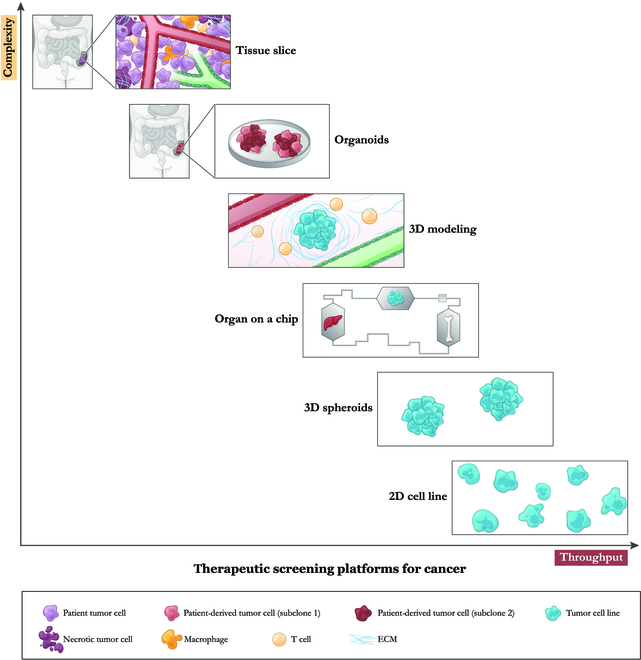
A schematic illustrating screening platforms for cancer shows different levels of complexity in modeling the TME and varying throughput for conducting drug screening.

**Table.** Comparison of 2D cell lines and 3D organotypic models of human cancers.

Model	Benefits	Limitations
2D cell lines	• Simple to establish and maintain • High-throughput screening capability • Easy downstream molecular analysis enables identification of intracellular pathways	• Lack 3D complexity of the TME • Many passages may induce genetic drift from the tumor of origin and fail to recapitulate the original tumor genetic heterogeneity
3D in vitro cultures	• Can be established in days to weeks • Facilitate study of tumor cell, stromal, immune cell signaling, interactions, and migration; model the physical/chemical microenvironment (ECM stiffness, oxygen/nutrient gradients) [13–20] • Facilitate flexible arrangement of components to tailor TME reconstruction to research interests • High-throughput screening capability	• Limited biological and disease relevance given simplified recapitulation of the TME [12]
Organs-on-a-chip and cancer-on-a-chip	• Physiologically relevant organ-level responses to drugs for screening/toxicity assessment; precise control of environmental cues, dynamic model of physiologic flow [23,31,32] • Application to pharmacokinetic and pharmacodynamic studies [30] • Capable to incorporate patient-derived organoids and tissue culture slices [28,29]	• Low-throughput screening capability due to time and resources required for design and engineering [32]
Tumor organoids	• Preserve the original tumor’s genetic and phenotypic characteristics • Exhibit drug sensitivity representative of interpatient, intertumoral and intratumoral heterogeneity; predict responses to targeted agents/chemotherapy [33,36,64,65] • Other model systems (e.g., mouse xenograft) can be incorporated to increase efficiency of organoid expansion [37] • Novel air–liquid interface method maintains immune populations, allowing for immunotherapy testing [39,40]	• Low-throughput screening capability due to scarcity of starting materials, time required to grow/expand culture • Predictive for only a subset of drugs/patients (e.g., predicted clinical sensitivity in patients with CRC receiving 5-FU + irinotecan but not 5-FU + oxaliplatin) [38,41] • Lack complete TME components, including immune cells, stromal cells, native ECM
Tumor slice cultures (TSCs)	• Clinic-friendly/personalized: established in <8 h, completed within 7 d • Replicate the original tumor; preserve tissue architecture, cellular composition, complex crosstalk between cells and tissue matrix, intratumoral heterogeneity while minimizing in vitro drift [42–44,50] • High-throughput readouts, including cell viability, cell death, histology, live imaging and molecular profiles (transcriptomics, proteomics) • Successfully established for drug testing of cytotoxic agents and small-molecule kinase inhibitors [45, 47,50–54,59]; concordance between in vitro TSC and patient responses (e.g., discrimination between chemotherapy-sensitive and resistant breast and CRC) [49,50,55,62] • Personalized prediction of clinical outcomes [61] • Preserve the intrinsic tumor immune microenvironment; facilitate immunotherapy testing predictive of in vivo patient responses [46,56–58,60]	• Require large tumor specimens; limit drug screening application to patients in whom systemic therapies are indicated following surgery (i.e., TSCs cannot be established in patients with unresectable disease) • Completed within 7 d and cannot be renewed in vitro, restricting the number of drugs that can be tested for a given tumor

With the introduction of cancer immunotherapy, the challenge to generate models that retain the human immune cell components has added a new level of complexity. The once popular patient-derived xenografts (PDX) using immune-deficient mice are no longer adequate given the lack of an intact immune system. Even with the engraftment of human immune cells into so-called “humanized” mice, immunocompetent PDX models remain difficult to generate and validate. Creating a platform that recapitulates the cellular and architectural complexity observed within a solid cancer is the holy grail in tumor modeling. The dynamic interaction between cancer cells and their neighboring immune and stromal cells has been shown to be a strong determinate of the tumor’s behavior. Thus, the spatial relationships, as well as the diversity of these components must be considered in order to generate observations that are predictive of the in vivo tumor response to chemical and cellular perturbations.

The current estimate of drug development success rate remains low. In a recent analysis of over 20,000 compounds that have reached the clinical phase, only 3.4% of oncology-related drugs were deemed “positive” from clinical trials [1]. One of the challenges in developing effective anticancer drugs is the ability to translate scientific discoveries from preclinical screening experiments to clinical trials. Many new therapeutics have shown promising results in early testing stages but have failed in later clinical stages, due to 3 major reasons. First and foremost, there often exists incongruity between preclinical models and the human cancers of interest, thus the screening platforms are poor surrogates of patient tumors [2,3]. Another important consideration is the suitability of the screening platform to test many different classes of therapeutics, from small molecules to antibody-based and finally to cell-based therapies. Lastly, stemming from an abundance of known and unknown off-target effects of the drugs, results from preclinical screens may lead to spurious findings that are not supported by underlying mechanisms. This review will highlight current more successful approaches in preclinical drug discovery and clinical testing, taking into consideration known complexities and limitations.

## Experimental Approaches for Cancer Therapeutic Screening

Cancer therapeutic screening during the preclinical stage plays an important role in developing novel therapeutics for effective cancer treatment. To better recapitulate the TME, drug screening platforms need to highly mimic tumor heterogeneity, including intratumor heterogeneity (within a tumor) that evolves over time, intertumor heterogeneity (between different tumors) among the primary site and the metastatic sites, and interpatient heterogeneity (between different patients) [4,5]. The heterogeneity of the tumor arises from genetic and epigenetic drift in the tumor cells along with changes in the microenvironment [6]. Tumor cells have a dynamic relationship with surrounding cellular components (immune cells, stromal cells, and endothelial cells), which dictate local physical and chemical cues, including extracellular matrix (ECM), chemokines, and cytokines [7–10]. These intricate and poorly understood interactions undoubtedly affect the tumor response to therapies and thus comprise a vital element of any drug development platform.

While no ideal system exists, certain characteristics and features are of paramount importance when choosing a tumor model for clinical testing. It is generally acknowledged that the greater the resemblance of the model to the original TME, the more likely it is for the results from the model to accurately reflect human disease. To that end, contemporary approaches aim to recreate the TME with its elements as it is in vivo, but the diverse cellular and extracellular heterogeneity in human cancers are difficult to fully appreciate, let alone to maintain in tumor models. Another challenge inherent in “personalized” oncology is the ability to make use of information from individual tumors to predict outcome. In order for such technology to be clinically relevant, the model must be generated within a short timeframe (e.g., within a week). Other desirable features of a drug sensitivity platform include the versatility to test a wide range of therapeutics from small molecules to antibodies and cells [11], while keeping the cost low in order to benefit the general public. Last but not least, the system should inform more than just a list of drugs and their sensitivities to a tumor but should also uncover underlying targets and mechanisms to improve our knowledge of the disease biology and pathogenesis.

Here, we will briefly review existing methods of drug testing and highlight the pros and cons for each, with the goal of understanding current limitations of new technologies that must be overcome in order to bring personalized oncology into reality (Fig. 1 and Table).

## 2D Cell Lines

Early experiments for drug screening started on 2-dimensional (2D) platforms by placing a layer of tumor cells using immortal cell lines on a 2D substrate, with or without drug treatment. This platform is simple to carry out with high-throughput screening capability. It allows researchers to identify a specific intracellular pathway that responds to a treatment of interest given that tumor cells can be easily collected for downstream molecular analysis. However, this platform falls short in capturing the 3-dimensional (3D) complexity of the TME. In addition, cell lines cultured in many passages could induce further genetic drifts from the tumor of origin and thus no longer recapitulate the original tumor genetic heterogeneity. Hence, drugs that can target the genetic mutation and subsequent signaling pathway of the cell line would likely to fail in cancer patients.

## 3D In Vitro Culture

To establish a 3D tumor model, cancer cell lines were used to form tumor spheroids with diameters on the order of hundreds of microns on nonadherent surfaces or using the hanging drop method. The 3D spheroids include tumor cell–cell contacts and model oxygen and nutrient gradients throughout. Spheroids can be formed in relatively short time (e.g., days to weeks) and allow for study of tumor cell, stromal, and immune cell interactions, offering a high-throughput platform when incorporated with automatic systems that handle spheroids.

Recent progress has been made to engineer 3D platforms that better recapitulate the TME and thus offer the potential for drug screening to target tumor cell and TME interactions [12]. Individual cancer cells or tumor spheroids embedded in 3D biomatrix—either synthetically made from biopolymers or type I collagen/matrigel derived from animals—allow for tumor cell–ECM interactions in 3D architecture, with the option to grow vasculature within the biomatrix for more sophisticated modeling of the TME [13]. The 3D platform allows for flexible arrangement of each TME component to reconstruct the TME based on the research interests. It has the advantage of precisely controlling one parameter at a time in the TME—either a cell type or a biophysical/biochemical cue—for drug testing, while mimicking the 3D nature of the TME.

This platform is excellent for testing therapeutics that target tumor cell–TME interactions in a 3D context, including tumor cell–ECM signaling [14], tumor cell epithelial-to-mesenchymal transition [15], and tumor cell migration and invasion [16], in parallel with modeling the physical and chemical microenvironment (e.g., varying the stiffness of the ECM, generating oxygen gradients or cytokine gradients, introducing fluid flows, etc.). In line with recent discovery in immunotherapy, 3D tumor platforms have also been developed specifically for studying tumor and immune cell interactions with application for immunotherapy [17,18], including immune checkpoint blockade screening [19] and chimeric antigen receptor-T cell therapy testing [20]. Although the 3D engineered platforms are advantageous in recreating components of the TME, they remain rudimentary compared to the native tumors that they simulate.

## Organ-On-A-Chip

Organ-on-a-chip is a recently developed technology that uses nano- or microfabricated devices to model the structural and functional complexity of human organs. Human cell lines are commonly used, though primary cells and human induced pluripotent stem cells are emerging, for constructing organs in the device chambers, including lung-on-a-chip, liver-on-a-chip, kidney-on-a-chip, gut-on-a-chip, and heart-on-a-chip [21,22]. The platforms provide organ-level response to drugs that are more physiologically relevant and suitable for drug toxicity evaluation when the liver compartment is included [23].

Cancer-on-a-chip (or tumor on a chip) is adapted from organ-on-a-chip concept and has become an attractive platform for anticancer therapeutic screening in cancer research. Microfluidics technologies using microfabrication or 3D bioprinting are utilized to build the compartments of the cancer-on-a-chip, and patient-derived cancer cell lines are used along with ECM components embedded in the microfluidic compartments to mimic the tumor and its 3D microenvironment. Progress have been made to establish different types of cancer-on-a-chip for therapeutic testing, including lung tumor chip, liver tumor chip, brain tumor chip, colorectal tumor chip, breast tumor chip, and pancreatic tumor chip [24–27]. Although cancer cell lines are often use to model the tumor, recently, patient-derived pancreatic organoids [28] and breast and prostate PDX tumor slice culture [29] that are more physiologically relevant were established in cancer-on-a-chip for successful culturing and subsequent therapeutic testing, highlighting the potential of cancer-on-a-chip as a preclinical therapeutic screening platform.

Multiorgans-on-a-chip that incorporate specific organs and tumors, which are connected by channels to interrogate and monitor dynamic interactions, have also been developed for pharmacokinetic and pharmacodynamic studies [30]. The advantage of multiorgan-on-a-chip can precisely control environmental cues and model the physiologic flow that creates dynamic interactions between the tumor and organs, while a majority of the current drug screening platforms are static nature. Toxicity evaluation is another critical component in preclinical drug screening; hence, organ-on-a-chip is becoming an important tool in this space [31,32]. Beyond technical considerations, a primary limitation of the organ-on-a-chip or cancer-on-a-chip model is the low- to medium-throughput nature of the platform, with capacity for at most dozens of replicates at any one time. Efforts are under way to create more automated, miniature organs-on-a-chip to increase throughput [32].

## Tumor Organoids

Primary cancer organoids—also known as tumoroids—are 3D cell cultures that are derived from human or mouse cancers. In contrast to the 3D culture of cancer cell lines described above, tumoroids typically refer to 3D spheroids originating from fragmented cancer tissues and are also known as patient-derived organoids (PDO). The platform has the advantage of preserving the genetic and phenotypic characteristics of the original tumor. In metastatic gastrointestinal cancers, it has been shown that PDO models had a positive predictive value of 88% in response to targeted agents or chemotherapy compared to patients in clinical trials [33]. Recent progress has been made in successful long-term culturing of cancer organoids in vitro and the capability to build large biobanks to store organoids, increasing accessibility and usability of organoids for preclinical therapeutic screening [34,35]. Kondo et al. [36] has summarized recent organoid platforms that have been used to screen 6 to 160 different drug compounds, with results demonstrating that organoid models exhibit various sensitivity to the compounds, representative of the interpatient, intertumoral and intra-tumoral heterogeneity of the tumors.

Limitations of PDOs as a drug screening platform include the low-throughput nature of the assay stemming from the scarcity of starting materials and the time to grow and expand the culture suitable for large-scale screening. To overcome this, the Inoue lab recently used patient-derived colorectal cancer (CRC) samples grown in mouse xenografts to expand the tumor mass as the basis for organoid culture. Incorporated with an automatic system to handle organoids, they reported the ability to screen 2,427 compounds and identify 15 hit drugs for subsequent drug testing, highlighting the potential of the organoid platform for high-throughput drug screening when applied thoughtfully and coupled with other methods. Growing patient-derived tissue in mouse xenografts can increase efficiency in organoid expansion compared to in vitro growth; however, one should note that genetic and phenotypic drift from the original tumor could occur after 5 cell passages [37]. There are powerful new approaches for screening at one time tens of thousands drugs, and their application to this system and other systems to be described will be fascinating.

Despite promising reports of its use, major limitations remain in implementing primary tumor organoids to predict the clinical sensitivity of human cancers. In a prospective, multicenter clinical study, Ooft et al. [38] reported the results of generating and testing PDOs in a cohort of CRC patients. The primary objective of the study was to identify non-responders to chemotherapy using in vitro testing of the “tumoroids” when compared to clinical outcome. Their overall success rate of generating tumor organoids was 63%, with the completion of drug sensitivity testing within a clinically relevant time frame of about 2 to 3 weeks. However, the results showed that the in vitro assay was predictive of clinical sensitivity only for patients receiving 5-fluorouracil (5-FU) and irinotecan but not 5-FU and oxaliplatin; both combinations are currently recommended as first-line therapy in CRC. The discrepancy in the ability of organoid testing to inform sensitivity for different drugs highlights our knowledge deficit in cancer modeling. Further improvements in organoid technology are needed to advance its clinical application, including the incorporation of other cellular components such as immune cells and stromal cells into the assay. Furthermore, the lack of tumor infiltrating immune cells in organoids poses a limitation on their use for testing immunotherapies. In recent work by Neal et al. [39], patient or mouse tumor-derived organoids maintaining the immune populations were successfully established using an air–liquid interface method, making it a suitable model for monoclonal antibody-based immune checkpoint blockade testing of immune–tumor cell interactions [40]. However, the current organoid models still lack other components of the TME, including the stromal cells and native ECM, which are known to influence tumor cell response to therapeutics. Not surprisingly, when PDOs were screened for sensitivity to a panel of “off-label”/investigational drugs in a cohort of CRC patients on standard chemotherapies who experienced tumor progression, the technology failed to predict clinical response [41]. Again, the study highlighted several limitations of the technology, including the relatively low rate of success in generating organoids (i.e., 57% in CRC, 17% in non-small-cell lung cancer), the time delay from testing to drug treatment (~3 to 4 wk), and the limited number of drugs screened.

## Tumor Slice Cultures

Organotypic tumor slice cultures (TSCs) are thin tissue slices precisely cut from patient tumor samples or mouse tumors, with thickness ranging from 100 to 400 μm and diameters in the order of several millimeters. The thickness of the tumor slices is optimized for sufficient nutrient and oxygen diffusion to sustain in vitro culturing while maintaining the intact TME. The key feature of TSCs is their ability to retain intratumoral heterogeneity, including tumor cells, stromal cells, immune cells, vasculature, and ECMs. Indeed, TSCs are direct replicates of the original tumors from which they are cut, thus providing a more relevant in vitro model platform for drug testing [42–44]. An abundance of information about the TME can be gleaned from TSC given the tumor- and cell-level detail afforded by this robust platform as well as the numerous high-throughput readouts, including cell viability, cell death, histology, and live imaging, along with molecular profiles from bulk to single-cell transcriptomics and proteomics [45–51]. More recently, the application of spatial “omics” analyses further expands the utility of TSCs to explore the dynamics of tumor response to drugs and other experimental manipulations. This form of 4-dimensional biology will undoubtedly improve our understanding of cancers and their treatment options.

With improvements in tissue handling and culture conditions, tumor slices can be readily prepared and grown in vitro for days to months without marked change to their morphology or architecture. In most cases, cultures can be used for drug testing as early as the day following slicing to allow for “recovery” from the trauma. The immediate availability of the slice platform offers 2 major advantages over other systems: (a) dramatically shortened assay time and (b) maintenance of tumor heterogeneity while minimizing in vitro “drift” or selection. Whereas the development and testing of PDOs in expert hands can be completed in ~3 weeks—which is very respectable and quick compared to that of tumor cell lines or PDX mice—organotypic culture-based drug testing can be completed within 7 d, making it much more clinic-friendly as a “personalized” sensitivity test. During the few days of drug testing, there is negligible time for in vitro selection to take place, thus preserving heterogeneity. Tumor slices, prepared from either human tumors, PDX tumors, or mouse tumors, have been successfully established for drug testing using cytotoxic agents and small-molecule kinase inhibitors [45,47,50–54]. Interestingly, tumor cells embedded within the TME have been shown to respond differently to anticancer drugs compared to in vitro models such as organoids derived from the same tumors.

A number of reports indicate that organotypic slice cultures can be established using patient-derived tumors, and the results of drug sensitivity testing suggest close concordance between in vitro and in vivo responses in patients. For example, breast cancer TSCs established by Naipal et al. [49] enabled discrimination between chemotherapy-sensitive and chemotherapy-resistant tumors based on morphologic examination, cell proliferation, and analysis of apoptosis. In CRC TSCs, Sonnichsen and colleagues [55] treated slices with different concentrations of 5-FU and a modified 5-FU and oxaliplatin (FOLFOX) regimen to assess drug susceptibility of individual tumors and demonstrated a range of responses that correlated with patients’ outcome. Gerlach et al. [50] similarly found drug- and dose-dependent variations in apoptosis and cell loss in chemotherapy-treated head and neck squamous cell carcinoma TSCs. The authors commented that an advantage of TSC, beyond the ease of establishing culture, is the preservation of cellular composition, complex crosstalk between cells and the tissue matrix, and thus more accurate assessment of treatment susceptibility .

More recently, investigators have exploited the organotypic culture to monitor the immune responses to various manipulations within the intact human cancers. Sivakumar et al. [56] prepared tumor slices from several syngeneic mouse models and PDX tumors with infiltrated immune cells for immunophenotyping and demonstrated the immune landscape and populations within the tumor slices, which were representative of the primary tumors. Of note, the immune cell composition in slices matched corresponding tumor cores on multicolor flow cytometry analysis. Extending this to human pancreatic adenocarcinoma (PDA), Jiang et al. [46] found that precision-cut tumor slices remain stable in culture for at least a week and retain tumor infiltrating T cells, macrophages, and stromal cells while capable of responding to drugs and allowing carboxyfluoroscein succinimidyl ester-labeled lymphocytes to migrate into the tumor slices. They went on to test PDA tumor slices with the combination of immune checkpoint inhibitor programmed cell death (PD-1) and C-X-C chemokine receptor 4 receptor blockade, showing that the drugs significantly enhanced reactivation of the endogenous CD8^+^ T cell antitumor function [57]. Specifically, live 3D confocal fluorescence imaging captured real-time migration of CD8^+^ T cells from peritumoral stroma toward cancer cells after combination treatment leading to tumor kill [57]. Notably, these in vitro results predicted findings from the COMBAT trial, a 2-cohort, phase IIa study in patients with metastatic pancreatic cancer treated with drugs targeting C-X-C chemokine receptor 4 and PD-1, motixafortide and pembrolizumab [57,58]. The clinical finding of a 34.5% disease control rate in chemoresistant PDA patients provides validation of the PDA TSC as a clinically relevant platform for drug discovery and testing.

Significant insights have been gleaned from studying organotypic tumor slices of microsatellite stable human colorectal liver metastases (CRLM), which are also resistant to immune checkpoint blockade. Jabbari et al. [59] investigated the response of CRLMs to conventional chemotherapies 5-FU, oxaliplatin, and irinotecan using a TSC model. Following in vitro exposure to the drugs, tissue slices were dissociated and subjected to single-cell transcriptome analyses to identify cell-type-specific responses to the treatments. While the sample size was small, they identified 2 patterns of response that correlated with the molecular subtypes of CRC. Tumors with the “basal/stem-like” type responded to 5-FU/irinotecan better than to 5-FU/oxaliplatin, which was associated with a down-regulation of PD-1 ligands and an activation of tumor-infiltrating T cells. Conversely, CRLMs that expressed T cell immunoglobulin and mucin domain-containing protein TIM-3 ligands such as galectin-9 were susceptible to the synergistic effects of anti-T cell immunoglobulin and mucin domain-containing protein-3 blockade in combination with chemotherapy. These studies involving modulation of the TME in human organotypic TSCs provided novel insight into the effects of chemotherapies in specific subtypes of CRLMs [59]. Such findings have therapeutic implications that could improve the outcome of CRLM by the selected use of chemo-immunotherapy. In another example, Sullivan et al. [60] used CRLM tumor slices to explore the role of interleukin 10 (IL-10) as a mediator of immune suppression. When anti-IL-10 blocking antibodies were exposed to tumor slices, they found significant tumor kill that was mediated by the reactivation of endogenous antitumor T cells. The authors deduced that tumor myeloid cells/macrophages are primarily responsible for the observed immune suppression. In addition, they found that IL-10 blockade potentiated the antitumor effects of carcinoembryonic antigen-specific chimeric antigen receptor-T cells in CRLMs. Together, these studies strongly support the use of human tumor organotypic cultures as models of cancers that retain the intrinsic microenvironment, including the immune components, and are suitable for immunotherapy research and testing. The ease of therapeutic manipulation and the diverse array of experimental readouts—from bulk assessment of viability to single-cell “omics”—make the platform versatile for many applications.

## Limitations of Organotypic Slice Culture

Despite decades of use in research laboratories, organotypic slice culture has not been popularized in clinical testing. Even though Majumder et al. [61] reported a high success rate in using human TSC to determine drug sensitivity in over 100 cancer cases, the technology failed to translate into clinical use. While the specific reasons are unclear, there are many variables that may be limiting the widespread adoption of organotypic cultures in personalized drug testing. The most fundamental question relates to the ability of the slice culture to respond to drugs in ways that are representative of the human disease. Numerous studies have highlighted the fidelity of tumor slices to capture and maintain the morphologic and architectural features of the original human cancers over the course of their ex vivo culture. The similarities extend across multiple biologic dimensions including gene expression and signaling pathways. However, large-scale studies comparing in vitro and in vivo responses to drugs are lacking. Nonetheless, multiple reports from small cohorts undergoing tissue-based testing are supportive of the underlying premise that the behavior of cancer slices correlates well with clinical response. For example, in our study of colorectal metastases, changes in viability of TSC following chemotherapy treatments (e.g., FOLFOX and FOLFIRI) were consistent with pre-op responses to the same drugs in 8 of 10 cases. Similarly, Zhu et al. [62] performed a prospective evaluation of glioblastomas treated with radiation and temozolomide following surgery. They found an 86% agreement between time to progression following treatment and ex vivo response to the same therapy using resected organotypic cultures. As noted above, in a large study conducted by Majumder et al. [61], a machine learning algorithm was developed based on an ecosystem built around tumor slices from 109 cases of colorectal and head/neck squamous cell carcinomas with the goal of predicting drug sensitivity. When applied to a test group of 55 patients, the algorithm correctly predicted the clinical outcome with a 100% sensitivity and 92% specificity. Collectively, these and other studies suggest that organotypic cultures of human cancers are faithful surrogates that reflect intrinsic biologic response of the disease and respond to drugs in a manner similar to that of the patient’s tumors.

One can thus only assume that the main limitations of using patient-derived tissues in clinical practice lie with implementation barriers. As briefly discussed, there are multiple methods of studying patient-derived materials, and none has reached the clinics. One popular approach is the creation of PDX mice using patient tumor materials, and large banks of such mice have been generated for many common human cancers such as breast cancer, lung cancer, prostate cancer, and CRC. These resources have contributed significantly to drug development and cancer research, but when it comes to serving as a tool for individualized drug testing, the PDX model fails to produce meaningful results due to the long latency (e.g., months), unpredictable take-rate, and associated costs [63]. Consequently, there are significant limitations related to “selection” and drift, leading to genetic and epigenetic deviations from the parental tumor. Moreover, PDX mice fail to recapitulate the immune microenvironment. Such deficiencies are challenging to overcome.

Perhaps the most utilized means of drug testing rests with PDOs, which are relatively efficient to create and have shown promise in their clinical correlations. While a number of commercial entities have offered assays that make use of clinical specimens from resected tumors to core needle biopsies, little is known about test performance in terms of prediction accuracy and patient outcomes in the real world. Despite multiple reports suggesting that treatment responses of PDOs in certain gastrointestinal cancers mirror clinical results [33,64,65], recent trials have shed light on the value and limitations of PDO as a clinical tool. In the prospective TUMOROID trial of 61 CRC patients, PDOs were generated from 40 of 63 biopsies (i.e., 63%) and testing was completed within 2 to 3 weeks of the biopsy. Analyses indicated that in vitro treatment sensitivity accurately predicted clinical response in 80% of patients receiving irinotecan-based chemotherapy but failed to predict response for those receiving oxaliplatin-based regimen [38]. This apparent discrepancy points to incomplete understanding of PDO drug testing. The authors remarked that the lack of the native microenvironment could be a contributing factor, but other nuances of drug interactions in combination therapies also deserve consideration. These drawbacks notwithstanding, PDO continues to hold promise as a clinical tool that deserves further technical refinement.

Organotypic slice cultures overcome some of the limitations of PDO including a high success rate (>90%) without latency and the preservation of the native TME. However, the major limitations of TSC are the inability to renew in vitro and the requirement for large tumor specimens. Consequently, this restricts the number of drugs or combinations that can be tested for a given tumor, and we believe in the future that combinatorial approaches to drug treatment are going to become increasingly important for effective tumor killing. Currently, slice cultures rely on the availability of surgically resected tumor specimens that are sufficiently large to undergo precision cutting and handling. In our hands, we typically make use of 6-mm punch biopsy cores to generate thin slices, while cores <3 mm in diameter are difficult to handle. This severely limits the application of the technology to a small subset of patients in whom systemic therapies are indicated following surgeries. In contrast, the patient population who would benefit most from drug sensitivity assays are those with unresectable disease awaiting some form of systemic therapy. Stemming from this practical limitation, clinical implementation of organotypic TSC for drug testing remains an elusive goal. Future efforts to develop novel technologies to overcome this shortcoming are necessary to advance the platform toward clinical adoption.

## Summary and Future Perspective

Organotypic tumor cultures offer a promising preclinical drug screening platform to better predict human cancer response to drugs because they allow researchers to monitor tumor cells within their native TME, which is currently lacking in other in vitro models. However, significant technologic advances are necessary to overcome the need for large tumor specimens as starting materials. Some of the sophisticated new tools of nanotechnology offer exciting possibilities for the procurement and the multiple assays of very small tumor samples. Another possibility would be the single-cell-analyses patient tumor cells and other cells from the native TME—searching for algorithms that would correlate with effective treatments. An ideal drug sensitivity assay would make use of biopsies that are readily accessible, while incorporating the TME to best reflect the behavior of the cancer in question. In designing the next generation of assay, it needs to take advantage of core needle biopsies no larger than 18-gauge needles. Importantly, drugs of interest should be exposed to tumor tissues that are least manipulated or selected in vitro, ideally within their native TME. However, following treatment, assessment of response should focus on the tumor cells as the primary readout, while changes to other cellular components intrinsic to the TME should be secondary. Other desirable features include the ability for high-throughput screening, short turnaround time, and low cost. Efforts are under way to create drug testing platforms with these principles in mind, including the use of microfluidics and machine learning tools [29,61,66–69]. If successful, patients will benefit greatly from selectively receiving treatments that have a high probability of clinical success, thereby minimizing toxicities and associated financial burden—a desired goal of “personalized” oncology.
